# Habitat Hydrology and Geomorphology Control the Distribution of Malaria Vector Larvae in Rural Africa

**DOI:** 10.1371/journal.pone.0081931

**Published:** 2013-12-03

**Authors:** Andrew J. Hardy, Javier G. P. Gamarra, Dónall E. Cross, Mark G. Macklin, Mark W. Smith, Japhet Kihonda, Gerry F. Killeen, George N. Ling’ala, Chris J. Thomas

**Affiliations:** 1 Institute of Geography & Earth Sciences, Aberystwyth University, Aberystwyth, United Kingdom; 2 Biomedical and Environmental Sciences Thematic Group, Ifakara Health Institute, Ifakara, Tanzania; 3 Institute of Biological, Environmental and Rural Sciences, Aberystwyth University, Aberystwyth, United Kingdom; 4 School of Geography, University of Leeds, Leeds, United Kingdom; 5 Vector Biology Department, Liverpool School of Tropical Medicine, Liverpool, United Kingdom; Institut Pasteur, France

## Abstract

**Background:**

Larval source management is a promising component of integrated malaria control and elimination. This requires development of a framework to target productive locations through process-based understanding of habitat hydrology and geomorphology.

**Methods:**

We conducted the first catchment scale study of fine resolution spatial and temporal variation in *Anopheles* habitat and productivity in relation to rainfall, hydrology and geomorphology for a high malaria transmission area of Tanzania.

**Results:**

Monthly aggregates of rainfall, river stage and water table were not significantly related to the abundance of vector larvae. However, these metrics showed strong explanatory power to predict mosquito larval abundances after stratification by water body type, with a clear seasonal trend for each, defined on the basis of its geomorphological setting and origin.

**Conclusion:**

Hydrological and geomorphological processes governing the availability and productivity of *Anopheles* breeding habitat need to be understood at the local scale for which larval source management is implemented in order to effectively target larval source interventions. Mapping and monitoring these processes is a well-established practice providing a tractable way forward for developing important malaria management tools.

## Introduction

There is a growing need to target malaria vector mosquitoes at their environmental resources through larval source management [1–8]. To implement such strategies effectively we need to be able to identify productive vector larval habitats [2,7,9–13]. Vector aquatic habitats are controlled by temporal and spatial hydrological dynamics [14] which need to be understood if habitat targeted interventions are to be successful [15,16].

Rainfall is a key determinant of malaria transmission [14], as it governs the availability of aquatic habitats required for breeding by vector mosquitoes. Despite this, observed relationships between rainfall and malaria transmission are variable [17] and poorly understood [18]. Recent advances in understanding of thermal drivers of malaria transmission [19–21] have not been matched by similar advances in our understanding of response to precipitation, despite this being the primary forcing climate variable in observed trends in malaria transmission in Africa over the last century [22].

Studies have demonstrated a link between habitat type and their ability to support vector larval populations [2,9,11,13,23,24]. However, such studies do not classify aquatic habitats according to the geomorphological and hydrological processes that control their formation and persistence [14]. This has led to inconsistencies when identifying the relative vector productivity of water body types. For example, Ndenga et al. [2] showed that the habitat type ‘puddles’ was the most productive, whereas Mutuku et al. [11] demonstrated that puddles are the least productive, both studies taking place in the western Kenyan highlands. Hydrologically speaking, a puddle is an ambiguous term as they can form and persist due to a number of different hydrological processes. For instance, pluvial puddles that are rainfall fed will be vulnerable to evaporation and may not provide productive habitats, whereas puddles that form due to rising water tables may persist for a longer period of time and may therefore be more productive. In this sense, the two puddles are distinct in terms of their dynamics and their responses to meteorological conditions, and should be classified accordingly.

Do Manh et al. [25] also examined larvae in different water body types for a rural area in Vietnam. However, these water body types were classified by land use, with no consideration of their geomorphological setting and hydrological controls. For instance, ‘ground pools’ included buffalo wallows, borrow pits, natural depressions, fish ponds, and manmade drains but these habitats are controlled by different hydrological processes. Borrow pits are likely to be fed by localised direct runoff, whereas permanent fish ponds are likely to exist where water table levels remain at the surface or where springs allow the pool to exist independently of rainfall [14]. 

In northern Angola a negative relationship was found between malaria transmission and distance to rivers [26]. This study was conducted in the dry season but the importance of river channels for supporting productive vector habitats can vary throughout the hydrological year. Specifically, large perennial rivers with seasonally inundated floodplains can support a number of productive vector habitats shortly after the wet season, such as the Gambia [27] and the Nile in Sudan [28]. Whereas the cessation of river flow in ephemeral channels during the dry season can produce chains of shallow pools [14] providing productive vector habitats [29] but will be prone to flushing out during the wet season due to fast flowing water [30,31].

To improve our understanding of vector larval habitats, it is important to determine the geomorphological and hydrological processes that govern the formation of vector aquatic habitats [14]. Ignoring these can lead to misinterpretation of the influence of rainfall patterns on malaria transmission [17] which currently forms the basis, along with other environmental components including temperature and humidity, for disease mapping and modelling [14]. 

Earlier studies have shown the potential for linking hydrological process based understanding to malaria [32] and mosquito dynamics [33,34]. The aim of this study is to expand this approach to the landscape scale by linking geomorphological and hydrological processes with malaria vector habitat productivity within a large sub-catchment (200 km^2^). This was achieved by monitoring *Anopheles* larvae over a 12 month period across a range of aquatic habitat types classified according to their geomorphology and hydrology and comparing them to changes in rainfall, river stage and water table level.

## Methodology

### Ethics

Ethical approval was granted by the National Institute for Medical Research, Tanzania, and Ifakara Health Institute's Review Board. Before larval sampling, verbal consent was requested from land owners and residents before entering fields or crossing compounds.

### Study Site

**Figure 1:**
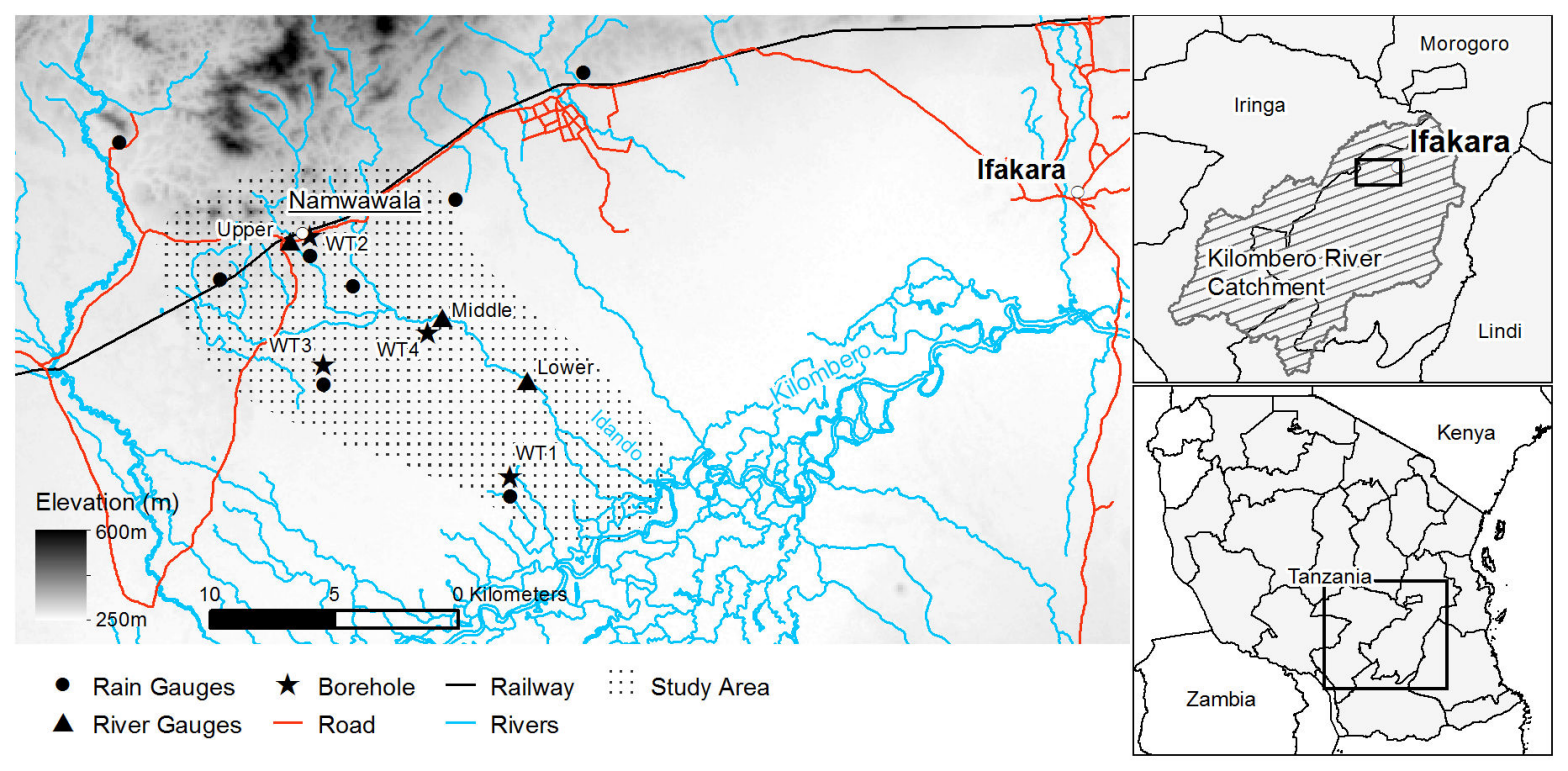
Kilombero Valley study area. The location of hydrological monitoring instruments is shown that recorded rainfall, river stage and water table depth over a 12 month period. Background elevation data is provided by the Shuttle Radar Topography Mission (SRTM) with a 90 m grid resolution.

The Kilombero River has a drainage area of 31,700 km^2^ (Figure 1) and is one of the principal tributaries of the Rufiji River, the largest river catchment in Tanzania. The Kilombero Valley is located within an asymmetrical half-graben between 30-40 km wide and 200 km long. The floodplain lies between 210-250 m.a.s.l. and is flanked by the Udzungwa Mountains (maximum elevation 2580 m) to the north and the Mahenge Highlands (maximum elevation 1520 m) to the south [35]. These upland areas receive over 1400 mm rainfall annually and the Kilombero Valley receives over 1000 mm [36] which is usually divided into two rainy seasons. Short rains occur in December and January with the main rainy season extending from March through to May [37]. The Kilombero Valley, one of the best characterised malaria transmission systems in Africa, had some of the highest reported historical rates of malaria transmission [38]. It is also one of the most advanced examples of successful transmission control in an African context, with near-elimination of *Anopheles gambiae* sensu stricto [39], the most historically important malaria vector locally [38] and across much of Africa [40], following the successful scale up of long-lasting insecticidal nets [41].

The focus of this paper is a 200 km^2^ area surrounding the village of Namwawala located 30 km to the west of Ifakara (Figure 1). The landscape is generally flat with hilly terrain to the north of the study area. The study area is drained by the seasonal Idando River which is typically 10 m wide and 2-3 m deep and is fed by two smaller tributaries at a confluence 5 km downstream of Namwawala. 20 km south of Namwawala lies the Kilombero River which flows throughout the year. During particularly wet years the Kilombero inundates the lower 7 km of the Idando sub-catchment but this did not occur during the sampling period of the present study. A majority of the local population are subsistence farmers [42] cultivating rice and corn without the aid of irrigation. Extensive burning of arable land takes place during the dry season to prepare the land for the planting of crops during the short rains which are harvested after the long rains in June [43]. 

### Hydrological monitoring

Rainfall was measured using a network of tipping bucket rain gauges. To account for the spatial variation in rainfall [44], eight rain gauges were positioned throughout the study area, ensuring a good geographical spread at a range of elevations (Figure 1). River stage was recorded using three vented pressure transducers positioned along the length of the Idando River. The upper gauge was located in the village of Namwawala; the middle gauge after the confluence of three tributaries capturing a large proportion of the water that leaves the Idando sub-catchment through the river channel system; the lower gauge further downstream, 7 km from the main Kilombero River which, during particularly wet seasons, can flood, pushing water back up the Namwawala tributary. Water table depth was recorded in four shallow (< 3 m depth) boreholes manually drilled into the soil.

### Entomological sampling

#### Water body type

**Figure 2:**
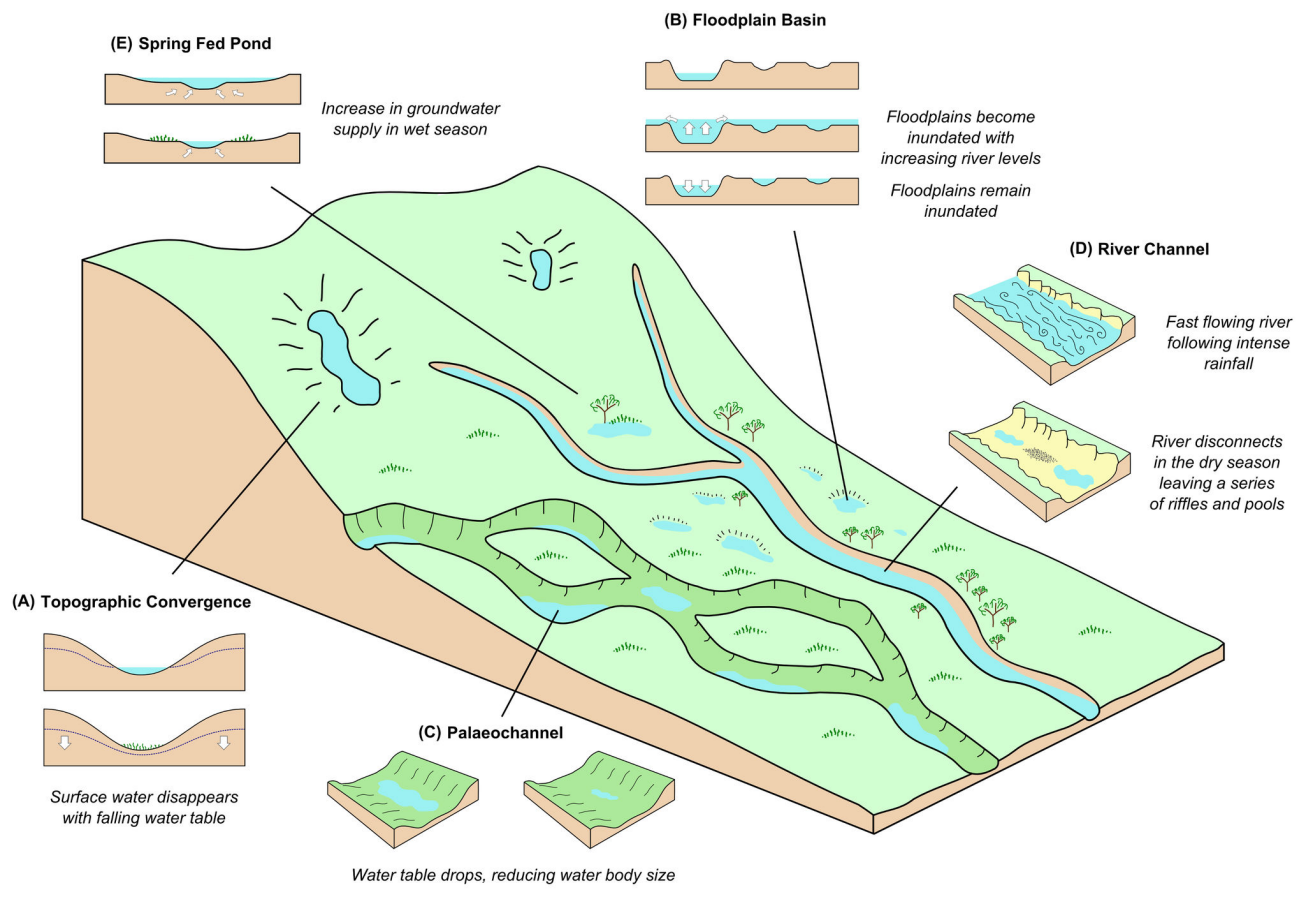
Diagram showing the different water body types. Included is a description of the key hydrological processes taking place in the dry and wet seasons within each water type found in the Namwawala area, Kilombero Valley, southern Tanzania.

**Figure 3:**
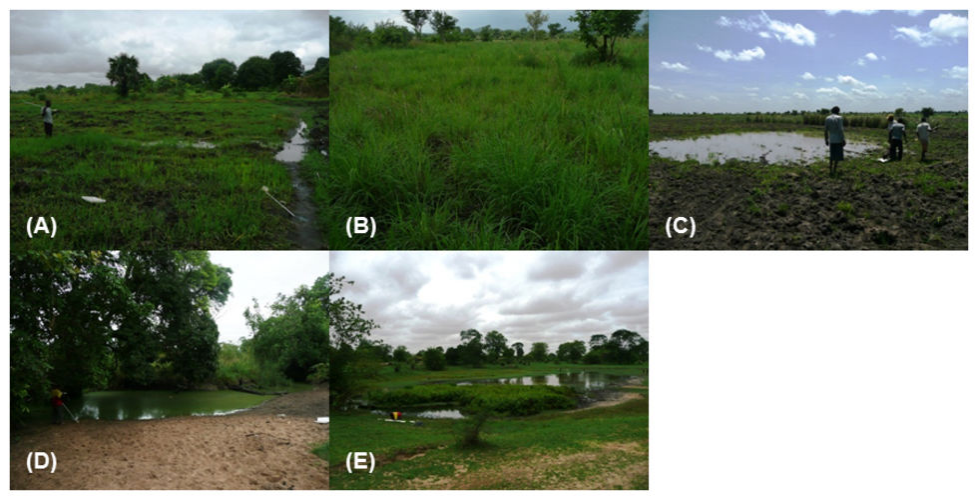
Examples from each water body type. The water body types were classified according to their geomorphological and hydrological characteristics. (A) Topographic convergence: saturated areas driven by topographic convergence of subsurface moisture; (B) Floodplain basins: depressions within floodplains of active river channels with well-developed levees; (C) Palaeochannels: associated with relict palaeochannel systems; (D) River channels: pools located in perennial or seasonally active river channels; and (E) Spring-fed pools.

Potential malaria vector habitats within the landscape were classified by their geomorphological and hydrological setting according to the classification scheme following Smith et al. [14]. Figure 2 provides a summary of the different water body types identified in the Namwawala area. Below is a description of the hydrological mechanisms that control surface water availability within the water body types and their potential for providing a vector habitat. A photograph of each water body type is provided in Figure 3.

- **Topographic convergence** water bodies represent areas of subsurface moisture accumulation [14]. Typically, such areas include valley and gully bottoms in small (< 1 km^2^) zero order catchments that do not have well developed channel networks. These are located in the hilly terrain in the north of the study area where rising water tables may intercept the surface resulting in surface ponding. This mechanism has previously been shown to be an important driver of vector habitat development in areas such as the western Kenyan highlands [16,45–49].
- **Floodplain basins** are shallow depressions lying close to river channels, particularly those with prominent, natural levees. These are inundated when river levels exceeds the height of the river banks and overtop levees. Some studies have found this to be a key process for the generation of vector breeding habitats. Notably, Bøgh et al. [27] found that most breeding habitats of *An*. *gambiae* *sensu* *lato* in the Gambia were generated by this mechanism. Similarly, Ageep et al. [28] showed that habitats supporting *An*. *arabiensis* in an area of northern Sudan were mainly driven by overbank flooding from the River Nile.
- **Palaeochannels** are sinuous linear depressions marking abandoned river channels that are no longer connected to active river channels. If the water table is sufficiently high, these depressions become saturated in a process similar to water bodies in areas of topographic convergence. During particularly wet years palaeochannels may reactivate with flowing water [14].
- **River channel** water bodies are located within river networks. During the dry season river levels can decrease sufficiently for the river to stop flowing. This forms a series of disconnected pools along the river channel, whose location are controlled by the topography of the channel bed [14]. These pools have been identified as a source of malaria vector habitats in a number of studies [11,50,51]. For instance, van der Hoek et al. [51] found the majority of vector habitats in Sri Lanka to be associated with pools formed in streams and river beds. During the wet season, river levels increase and pools reconnect causing water to start flowing within the channel. Larvae of most *Anopheles* species can only tolerate still or slowly moving water and are therefore vulnerable to high river flows, highlighted by the modification of channels to augment water flow as a larval control method [4].
- **Spring-fed pools** are water bodies fed by groundwater recharge and can persist throughout the year, independently of rainfall. This makes them important for sustaining vector populations through the dry season when many other water body types are likely to dry up [52–54]. Specifically, this provides a potential habitat for species that prefer permanent water bodies such as *An*. *funestus* [53].

**Figure 4:**
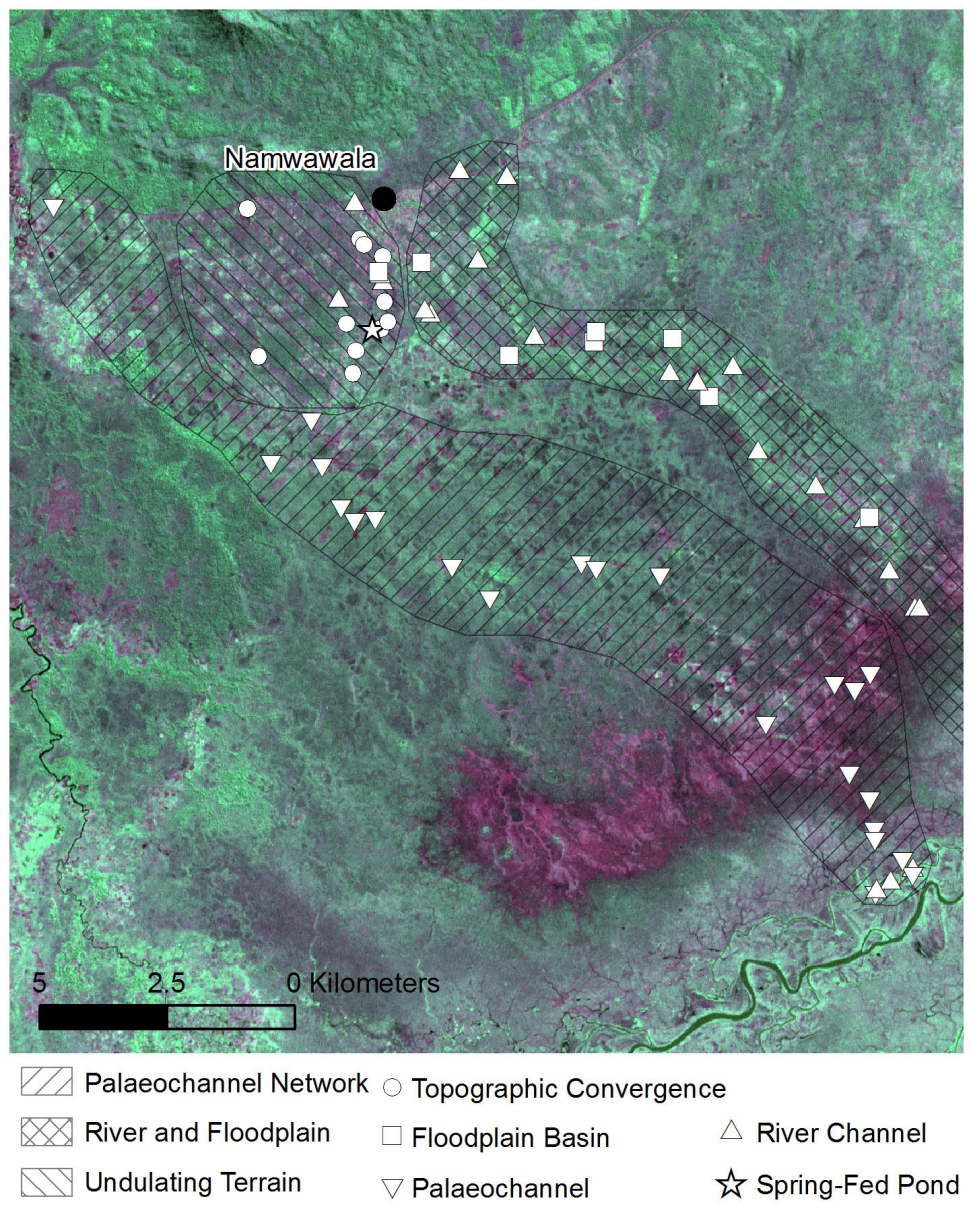
Larval sample locations categorised by water body type. The background image was captured on 10^th^ July 2001 by Landsat Thematic Mapper. The image is displayed as a false colour composite (red = band 7, green = band 5 and blue = band 4) with bright green indicating developing vegetation, dark green indicating mature or sparse vegetation, purple indicating bare soil and black representing water.

Landsat satellite imagery acquired on 10^th^ July 2001 (30 m grid resolution) was used to identify the main river channels and their floodplains (Figure 4). This imagery was also used to identify palaeochannels which appear as distinctive sinuous linear features either infilled with fine grained sediment (silts and clays) and organic matter that retain moisture making them appear darker than the surrounding landscape or comprising sandy deposits that form levees making them appear bright. 

A Digital Elevation Model (DEM) was extracted from 50 cm stereo Worldview satellite imagery acquired on 12^th^ February 2012 using standard photogrammetric techniques [55]. This was carried out using the DEM extraction tools within the image processing software ENVI [56] producing a DEM with a grid resolution of 2 m and a vertical accuracy of approximately 2 m [55]. This was used to identify areas of topographic convergence which have potential for the accumulation of moisture [16]. The DEM was also used to identify low-lying areas adjacent to river channels where flooding might occur. The features identified using the imagery and DEM were checked using field observations. There was also a single groundwater spring in the study area. This was not evident in the remotely sensed data and was mapped in the field. 

#### Water body sampling

The landscape was divided into distinct geomorphological zones through manual interpretation of Landsat imagery and DEM data and a mapped groundwater spring. Random stratified sampling was used to distribute 65 sample locations within these zones (Figure 4) in proportion to the observed frequency of each water body type in the study area. All locations were situated within 3 km of an occupied house, within the typical flight range of female *An. gambiae* [57]. Eight points were located in floodplain basins, eleven points were located in areas of topographic convergence, and only one located in the groundwater spring. Numerous habitats, however, were located in seasonally active river channels (22) and depressions within palaeochannel systems (23).

Sample locations were determined by field visits during the dry season in September-October 2011. At the first field visit to each location, the closest standing water body within a 150 m radius was selected as the sampling location, and its position recorded using a handheld Garmin Etrex GPS receiver with a horizontal accuracy of approximately 5 m. If a water body was not found within the search area the sample location was centred in an area where a water body was most likely to occur. This was determined by looking for depressions in the local terrain and identifying features such as caked mud, the presence of hydrophilic vegetation and dried hoof prints representing a potential watering hole for cattle.

Each location was visited 13 times from November 2011 to October 2012 at a frequency of approximately once every four weeks. At each visit, all water bodies within a 25 m radius of the location were identified and up to five water bodies were selected at random for surveying and their location recorded using the handheld GPS. A description of the site was taken, including the width and length of the water body. This was used to estimate habitat size by calculating the area as an ellipse and taking the outer 50 cm to represent the shallow edges of the water body where larvae tend to occur [23]. 

A purposive dipping strategy was employed [7,24] using a 350 ml dipper, whereby dips were made in places most likely to harbour larvae, such as around clumps of vegetation or protruding substrate, amidst floating debris, and along the periphery of the water body. The number of dips was decided *a priori* based on the size of the water body to be surveyed. A minimum of 10 dips were taken at each water body with the number increasing up to 40 for large water bodies (> 40 m in length). Other studies have adopted the use of sweep nets to determine the abundance of larvae [2] but the dimensions of these nets exceed the size of small scale aquatic habitats, such as hoof prints, at the fringes of larger pools of water.

Each dip was examined in a white plastic tray. Anopheline and culicine larvae were differentiated macroscopically based on body position and morphology [54]. Counts were made of early (1^st^-2^nd^ instars) and late-stage (3^rd^-4^th^ instars) anopheline larvae [58]. Where the total number of larvae caught in all dips at a water body exceeded 10, a random sample of 10 larvae was taken and specimens were stored in separate 1.5 ml eppendorfs in 98% ethanol for subsequent molecular species identification. Where the total number of larvae per water body was 10 or fewer, all larvae were taken for species identification. Pupae were not counted because anopheline pupae cannot readily be morphologically distinguished from culicine pupae in the field [2,54]. 

Genomic DNA was extracted from individual larvae and the amplification of ribosomal DNA was made using a multiplex polymerase chain reaction (PCR) for identification of the four sibling species of the *Anopheles gambiae* complex (*An. arabiensis*, *An. gambiae* s.s., *An. merus* and *An. quadriannulatus* ) [59]. Unamplified DNA was tested by a further PCR assay with the capacity to identify five species of the *Anopheles funestus* group including *An. funestus* s.s., *An. leesoni, An. parensis, An. rivulorum* and *An. vaneedeni* [60].

### Data analysis

#### Hydrometric data

Daily total rainfall was calculated for each rain gauge. Pairwise relationships between the gauges were analysed using Spearman rank correlations. The gauges were used to calculate areal average rainfall for the study area. Hourly water table depths were calculated by subtracting recorded water depth from the depth of the pressure transducer below the surface. 

#### Entomological data

In order to focus on indicators of habitat quality for malaria vectors, the number of late instar *An. arabiensis* larvae per dip was estimated [61–63]. Analysis was restricted to *An. arabiensis* as this was the only primary malaria vector species found in sufficient numbers (Table 1). Estimated numbers per dip and confidence intervals were derived using Generalized Estimating Equations (GEE) over the total number per dip of late instar stage anophelines and the proportion of *An. arabiensis* found in the PCR samples via bootstrapping of a mixture distribution. Analyses were performed with the geepack package [64], and the boot package [65] for R [66]. Contrasts in the number of late instar *An. arabiensis* larvae per dip between water body types were calculated using the Method of Variance Estimates Recovery (MOVER) [67]. A detailed description of the statistical analyses of entomological data can be found in the Methods S1. 

**Table 1:** Total *Anopheles* species count gathered throughout the sampling period and relative proportions.

	**Count**	**% of total**
***An. gambiae* s.l. complex**		
*An. Arabiensis*	503	25.2
*An. gambiae* s.s.	0	0
*An. Merus*	0	0
*An. Quadriannulatus*	0	0
***An. funestus* group**		
*An. funestus* s.s.	37	1.9
*An. Leesoni*	1	0.1
*An. Parensis*	0	0
*An. Rivulorum*	12	0.6
*An. Vaneedeni*	0	0
**Non-amplified specimens**		
*All*	1445	72.3
**Total**	1998	

It is important to note that the above methods make a number of inferences that must be acknowledged. The quality of the data was not optimal owing to the presence of zeros in larval numbers, inaccessibility of some locations during the wet season, over-dispersion, unbalanced surveys, and lack of knowledge regarding the covariance structure of the residuals. As a result, our analysis is free from a time dependent structure accounting for time correlations in the number of larvae. However, as a precautionary measure, our sampling dates were set at periods long enough to minimise causality due to autoregressive processes (i.e. periods longer than a generation).

The bootstrap estimated number of late instar An. arabiensis larvae per dip, including upper and lower 95% confidence intervals, were multiplied by the total area of available habitat per water body type, based on field observation of water body dimension per sample round, to derive an area-weighted abundance estimate of late-stage An. arabiensis larvae. This was compared with the hydrometric data, aggregated to monthly time steps to match the entomological sampling frequency, using Cross Correlation Functions in R [66] taking into consideration lagged relationships. Due to the highly variable nature of the larval data autoregressive time series analysis was not possible; this would require a much larger number of sample locations. 

## Results

### Hydrology

**Figure 5:**
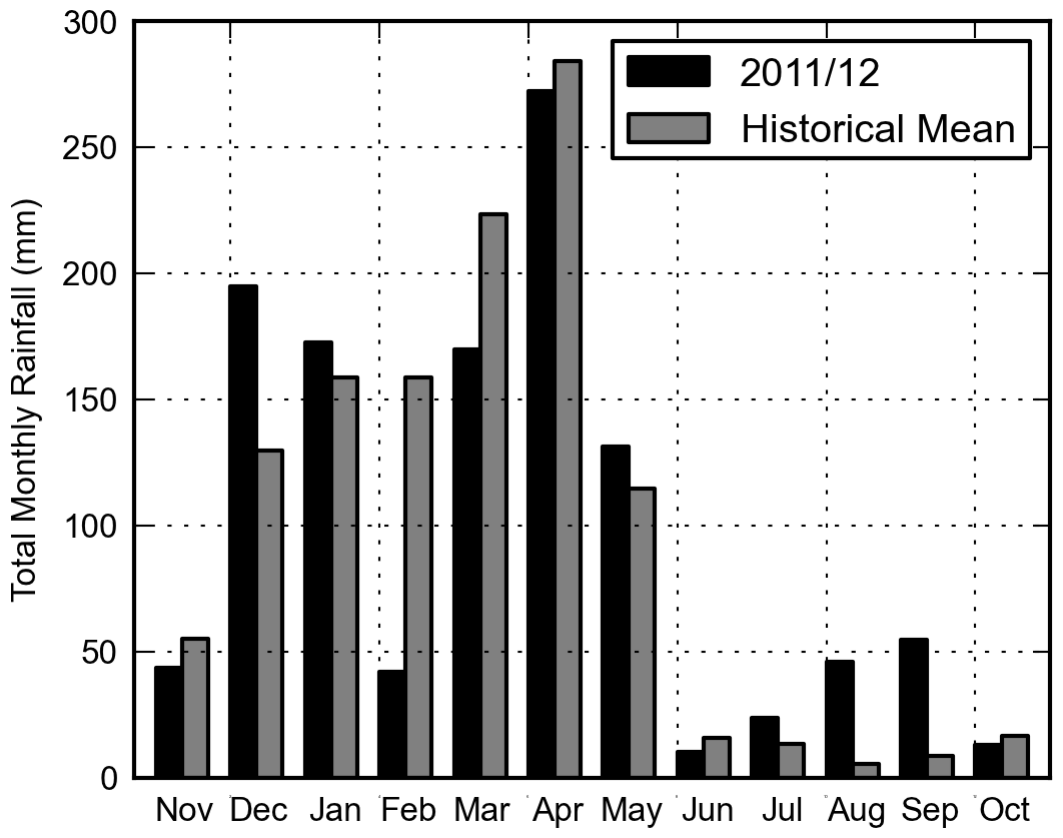
Total monthly rainfall. Rainfall was recorded using a network of eight tipping bucket rain gauges positioned throughout the study area. Historical mean (1969-2010) is calculated using rain gauge measurements recorded near the town of Ifakara located 30 km east of the study area.

**Figure 6:**
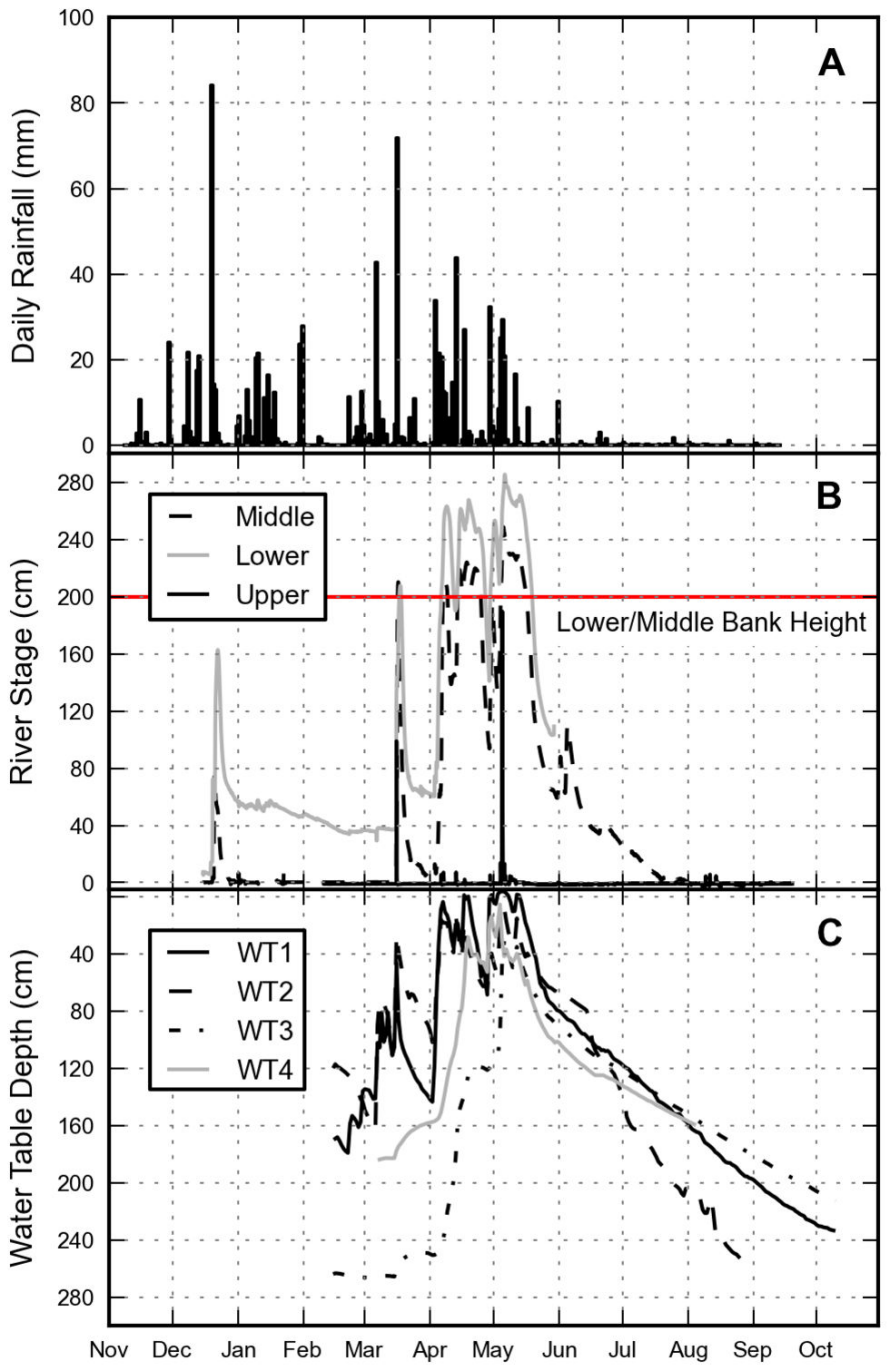
Graphs showing (A) areal averaged rainfall from eight gauges distributed throughout the study area summarised as daily totals; (B) mean hourly river stage height with bank level reference line for the middle and lower gauges; and (C) mean hourly water table depth below the surface.

The study area received a total of 1175 mm rainfall over the 12 month study period compared to a historical (1969-2010) annual average of 1186 mm recorded at a gauge near Ifakara. December was wetter than average and February was considerably drier receiving over 100 mm less rainfall compared to the historical average (Figure 5). Total monthly rainfall masks the intensity of individual rainfall events. Most notably, over 40% of the rainfall in both December and March occurred over a 24 hour period on 19^th^ December and 16^th^ March, respectively (Figure 6A). 

Precipitation was not distributed evenly over the study area. For instance, on 19^th^ December 131 mm was recorded at one rain gauge and just 18 mm was recorded at another less than 15 km away. Pairwise Spearman rank correlations showed that daily rainfall totals recorded at one pair of gauges were not significantly correlated (p = 0.52). The gauges were located only 20 km apart with a difference in elevation of less than 20 m. Rainfall recorded at all the other gauges were significantly correlated (p < 0.01).

River stage rose rapidly in response to rainfall (Figure 6B), particularly following intense rainfall events in December 2011 and March 2012. For instance, following the 16^th^ March rainfall event the stage at the upper river gauge rose from 0 cm to 115 cm and fell to 4 cm over a period of four hours. Further downstream at the middle and lower gauges persistent rainfall kept stage heights above zero from April through to mid-July. During April and May 2012 the stage height exceeded the height of the river banks at the middle and lower gauges leading to overbank flooding. During this period, the water table remained high (Figure 6C) with one gauge positioned close to the Kilombero River recording negative depth values indicating that water was pooling at the surface. 

### Entomology

Of the 1998 larvae taken for species identification, a majority were unamplified in the PCR process (Table 1) and were likely to be other species of *Anopheles* which are not malaria vectors (PCR tested for all significant vectors in the region). No An*. gambiae* s.s. larvae were found and less than 2% were identified as *An. funestus*. *An. arabiensis* made up over 25% of the total count. Most of the specimens identified as *An. funestus* (33) were found in water bodies located within ephemeral river channels. These habitats persisted throughout the hydrological year as shallow pools in the dry season which connect during the wet season as flowing water. 

**Figure 7:**
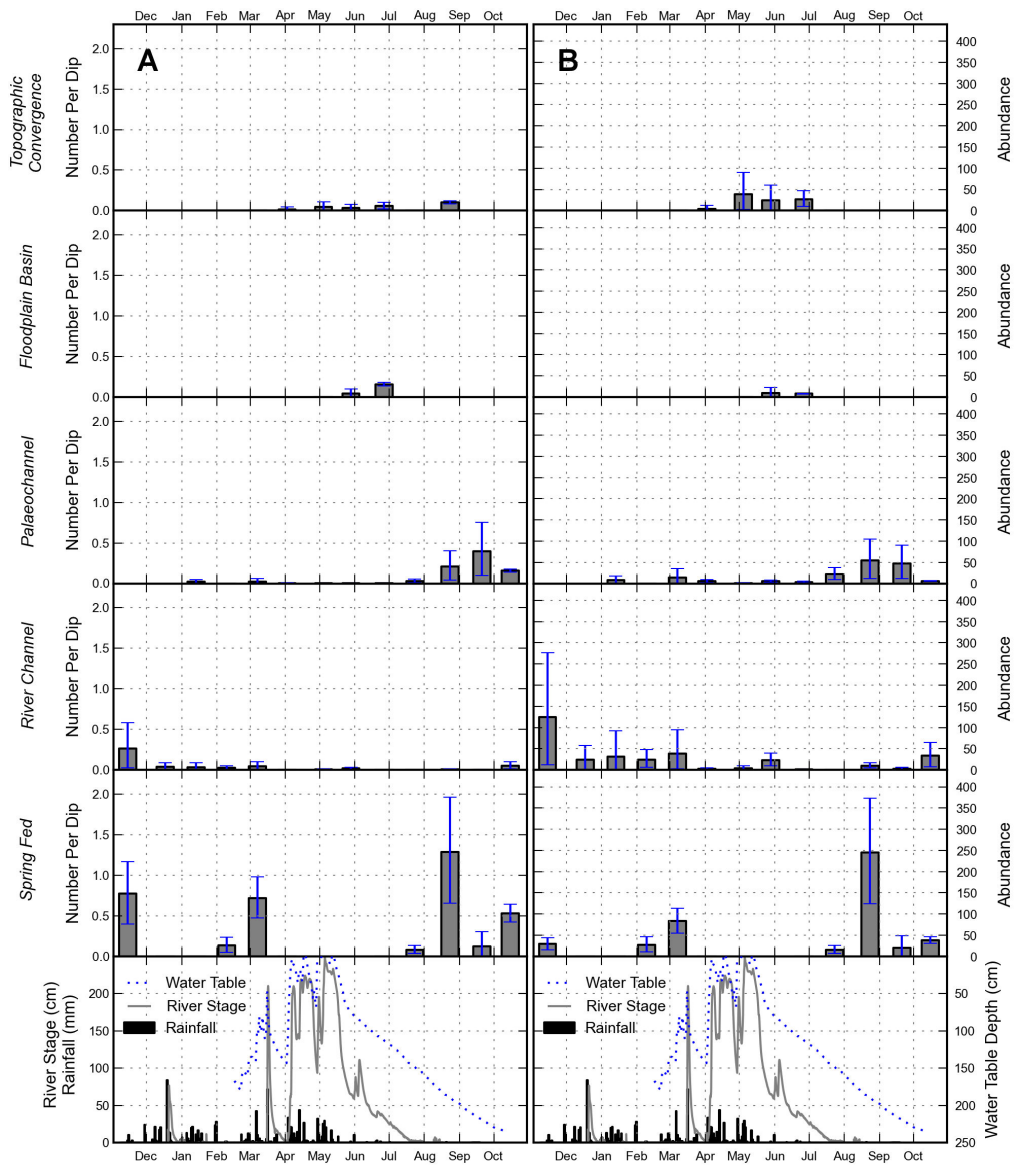
Plots of *An*. *arabiensis* estimates per water body type. (A) Bootstrap prediction estimates of late-stage *An*. *arabiensis* larvae per dip and (B) area-weighted abundance estimate of late-stage *An*. *arabiensis* larvae for each water body type. Area-weighted abundances and their 95% confidence intervals were calculated by multiplying estimated habitat size by the number of late-stage *An*. *arabiensis* larvae per dip estimated by bootstrapping a mixture distribution generated from GEE estimates of number of late-stage anophelines and the probability of finding *An*. *arabiensis* in the PCR samples. The hydrometric data is added for reference including hourly areal average rainfall, river stage recorded in the middle of the study site catchment and water table depth recorded towards the south of the study area.

The variation in estimated number of late-stage *An. arabiensis* larvae per dip (Figure 7A) and area-weighted abundance estimate of late-stage *An. arabiensis* larvae (Figure 7B) over the sampling period were similar suggesting that habitat size did not control the density of larvae found in each water body type. The abundance of *An. arabiensis* larvae increased in areas of topographic convergence from May to July 2012 following the peak of the long rainy season. Habitats within floodplain basins also showed an increase during this period following a peak in river stage which exceeded the bank level leading to flooding. River channel and palaeochannel habitats had background levels of vector larvae for most of the sampling period; however, both showed a reduction at the height of the long rains in April and May 2012. River channel habitats supported relatively high abundance of vector larvae over the dry season and short rains, from December 2011 to March 2012, when the river was not flowing, leaving a series of disconnected pools in the river bed. The spring fed pond was also shown to support high larval abundance during dry periods, most notably in August 2012. Despite this, very few numbers were found in the spring fed pond during the short and long rains. 

**Figure 8:**
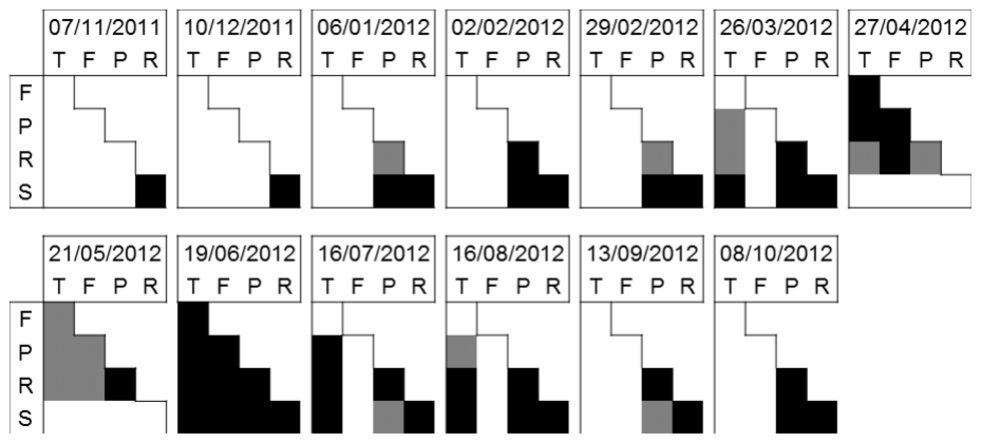
Contrasts in bootstrap estimated number of late-stage *An*. *arabiensis* larvae per dip using Method of Variance Estimates Recovery [67]. Black = significant difference (95% confidence), grey = no significant difference, blank = not available (due to absence of larvae in one or both habitat types). T = topographic convergence, F = floodplain basin, P = palaeochannel, R = river channel and S = spring-fed pond.

The estimated number of late-stage *An. arabiensis* larvae per dip in each water body type were shown to be variable over the sampling period (Figure 8). This was particularly true for the sample round immediately following the wet season (19^th^ June 2012), during which estimated vector larvae per dip were shown to be significantly different between every water body type. Throughout the sampling period the spring fed pond habitat type tended to be distinct from other water body types, with the highest larval densities in the study area recorded in four out of the thirteen sample rounds. 

Cross Correlation Function analysis showed that the area-weighted abundance estimate of late-stage *An. arabiensis* larvae across all the sites was not significantly related to the hydrometric data (Table 2). However, relationships existed when the abundance estimate was aggregated by water body type. Abundance estimates from areas of topographic convergence were positively related to river stage and were negatively related to water table depth reflecting a dependence on the wet season to raise water tables resulting in surface ponding. Floodplain basin abundance estimates were also related to river stage but with a one month lag. Here, flooding is widespread during the peak of the wet season but these water bodies only support vector larvae once the flood water has receded, due to infiltration and evaporation, to form smaller, shallower pools of water. Abundance estimates in palaeochannels had a positive relationship with river stage with a three month lag indicating that these habitats cannot support vector larvae during or shortly after wet periods.

**Table 2:** Correlation coefficients between the hydrometric data and area-weighted abundance estimate of late-stage *An*. *arabiensis* larvae per water body type.

	***0 lag***			***1 month lag***			***2 month lag***			***3 month lag***		
	***Rain***	***Stage***	***WT***	***Rain***	***Stage***	***WT***	***Rain***	***Stage***	***WT***	***Rain***	***Stage***	***WT***
***T***	0.62	0.97**	-0.7*	0.58	0.48	-0.39	0.03	-0.08	0.16	-0.06	-0.57	0.39
***F***	-0.99**	-0.25	0.1	0.24	0.78*	-0.42	0.53	0.29	-0.3	-0.25	0.03	0.06
***P***	-0.46	-0.47	0.59	-0.47	-0.28	0.25	-0.33	0.05	-0.14	-0.02	0.64*	-0.56
***R***	-0.28	-0.05	0.11	0.08	-0.07	0.15	-0.09	-0.19	0.26	-0.3	-0.15	0.27
***S***	-0.17	-0.2	0.33	-0.05	-0.27	0.11	-0.29	-0.11	-0.02	-0.05	0.61	-0.47
***All***	-0.35	-0.29	0.41	-0.09	-0.23	0.14	-0.26	-0.07	-0.01	-0.12	0.52	-0.41

Analysis carried out using Cross Correlation Functions. T = topographic convergence, F = floodplain basin, P = palaeochannel, R = river channel, S = spring-fed pond, WT = water table.Analysis carried out using Cross Correlation Functions.

Significant to Bonferroni adjusted confidence intervals at 99%** and 95%*.

Rain = rainfall, Stage = river level, WT = water table depth

T = topographic convergence, F = floodplain basin, P = palaeochannel, R = river channel, S = spring-fed pond, WT = water table.

## Discussion

Nearly three quarters of the larvae identified to species level were not *An. gambiae* s.s.*, An. arabiensis* or *An. funestus*, the major contributors to malaria transmission in Africa [39,41]. No An*. gambiae* s.s. were found and only small numbers of *An. funestus* were identified, whereas one quarter of the larvae tested were identified as *An. arabiensis*. The low densities of *An. funestus* are consistent with previous surveys of adult mosquitoes in Namwawala village [35,37,42,68]. The apparent absence of *An. gambiae s.s.* parallels observations in the adult population, reflecting the success of and long-lasting insecticidal net (LLIN) distribution programmes [39,41], suppressing anthropophagic species, such as *An. gambiae* s.s. and *An. funestus*, that are highly dependent on obtaining human blood indoors [69]. By contrast, *An. arabiensis* is not only more zoophilic, exophagic and exophilic, it also appears capable of safely entering and exiting houses containing LLINs even where and when it remained fully susceptible to their pyrethoid active ingredients [70,71], so this species can be described as being resilient to this vector control intervention [8,72]. 

A previous study has demonstrated an increase in vector population and subsequent malaria transmission at the height of the wet season [29]. However, this study found that overall area-weighted abundance estimate of late-stage *An. arabiensis* larvae fell across all habitat types during periods of prolonged rainfall associated with the height of the wet season in April and May 2012. This included a spring fed pond which is a permanent water body and is therefore assumed by the global index of malaria stability to be independent of seasonal fluctuations in rainfall [73]. Numbers of *An. funestus* were restricted almost exclusively to ephemeral river channels where water bodies persisted throughout the dry season as shallow disconnected pools, which reconnect in the wet season as a flowing river. This is consistent with observations of *An. funestus* behaviour showing them to have a preference for more persistent water bodies [53,74,75]. Although the precise location of such habitats may be difficult to predict, the ephemeral channels in which they are located are often mapped or can be readily identified in high spatial resolution (2 m) satellite imagery. Despite the availability of surface water throughout the hydrological year, no *An. funestus* were found in the spring-fed pond. Factors leading to this absence are uncertain and can perhaps be attributed to the relatively short study period leading to anomalous observations. However, environmental factors may also account for the absence of *An. funestus*, for instance the spring-fed pond is open and sunlit, whereas river channel habitats are characterised by overhanging tree canopies providing shade, a factor which has previously been shown to be significantly related to the abundance of *An. funestus* larvae [76].

Large-scale studies into climatic drivers of malaria transmission are often based on monthly aggregates of environmental data, including precipitation [14]. However, total monthly rainfall recorded in the Namwawala area over the sampling period masked the intensity of individual rainfall events, which can be an important indicator of a reduction in larval numbers due to the flushing out of habitats and displacement or death due to rain pounding [31,74]. This study found that daily measurements of rainfall are sufficient to capture these events. 

Area-weighted abundance estimates of late-stage *An. arabiensis* larvae were not significantly related to monthly aggregates of rainfall, river stage and water table. However, relationships became clear after the larval data was aggregated by water body type defined on the basis of its geomorphological setting and origin. For instance, a significant relationship existed between estimated vector larval abundance in areas of topographic convergence with water table depth, reflecting the topographic organisation of water in the landscape and the formation of pools following the wet season [16,45,46]. Vector larval abundance within floodplain basins was also related to river stage, but with a one month lag, representing the development of *An. arabiensis* vector larvae during the drying out phase of flood waters [28,77,78]. By contrast, vector abundances within palaeochannel habitats were related to river stage with a three month lag. This likely reflects the lack of dependence of *An. arabiensis* on this water body type during the height of the wet season. Again, the drying out phase of aquatic habitats appears to play a crucial role [28,77,79] with smaller, shallower and more turbid water bodies [74] providing dry season refuges in palaeochannels as the availability of water in other habitat types disappears, specifically floodplain basins and areas of topographic convergence.

Studies in areas such as the western Kenyan highlands have established relationships between hydrology and malaria vector numbers using terrain analysis because the distribution of water in the landscape is controlled by topography [16,45,46,49]. However, the present study site requires consideration of more than just topographical controls on hydrology including the influence of flowing water in rivers and palaeochannels, overbank flooding and habitats fed by spring water. We have shown that significant differences in vector larval abundance occur in habitats when they were classified by their hydrology and geomorphological setting. Furthermore, significant correlations existed between larval abundance and simple hydrometric data. This process based understanding can be used to model and forecast the spatial and temporal dynamics of malarial aquatic habitats. These findings should be incorporated into models of malaria transmission, particularly those that are limited to the influence of climate and weather on parasite and vector development [80–82].

The main finding of this study is that the spatial and temporal variation in malaria vector larvae can be explained according to the hydrological processes that govern the formation and persistence of different habitat types. Vector larvae productivity shifts to different water body types throughout the hydrological year in response to rainfall and subsequent changes in water table and river stage. Specifically, floodplain basins and areas of topographic convergence became dominant in the wet season with vector larvae retreating to palaeochannels, ephemeral river channels and a spring fed pond during the dry season. These dynamics are driven by hydrological and geomorphological processes, many of which can be mapped using remotely sensed data with the exception of spring-fed ponds which are reliant on ground mapping. This approach can provide valuable information for larval source campaigns for targeting productive habitats, particularly during the dry season.

## Supporting Information

Methods S1 **Description of the statistical analysis of entomological data: Generalized Estimating Equations and Method of Variance Estimate Recovery.**
(DOCX)

## References

[1] Ferguson HM, Domhaus A, Beeche A, Borgemeister C, Gottlieb M et al. (2010) Ecology: a prerequisite for malaria elimination and eradication. PLoS Med 7: 1-7. PubMed: 20689800. 10.1371/journal.pmed.1000303 PMC2914634 20689800

[2] Ndenga BA, Simbauni JA, Mbugi JP, Githeko AK, Fillinger U (2011) Productivity of Malaria Vectors from Different Habitat Types in the Western Kenya Highlands. PLOS ONE 6: e19473. doi:10.1371/journal.pone.0019473. PubMed: 21559301. 21559301 PMC3085476

[3] Gouagna LC, Rakotondranary M, Boyer S, Lemperiere G, Dehecq JS et al. (2012) Abiotic and biotic factors associated with the presence of Anopheles arabiensis immatures and their abundance in naturally occurring and man-made aquatic habitats. Parasites and Vectors 5. 10.1186/1756-3305-5-96 PMC3461495 22608179

[4] Imbahale SS, Githeko A, Mukabana WR, Takken W (2012) Integrated mosquito larval source management reduces larval numbers in two highland villages in western Kenya. BMC Public Health 12: 362-. PubMed: 22607227. 22607227 10.1186/1471-2458-12-362 PMC3433356

[5] Zhou G, Munga S, Minakawa N, Githeko AK, Yan G (2007) Spatial relationship between adult malaria vector abundance and environmental factors in western Kenya highlands. Am J Trop Med Hyg 77: 29-35. PubMed: 17620627. 17620627

[6] Killeen GF, Seyoum AK, Knols BGJ (2004) Rationalizing Historical successes of malaria control in Africa in terms of mosquito resource availabilty management. American Journal of Tropical Medicine and Hygiene 71: 87-93. 15331823

[7] Fillinger U, Lindsay SW (2006) Suppression of exposure to malaria vectors by an order of magnitude using microbial larvicides in rural Kenya. Trop Med Int Health 11: 1629-1642. doi:10.1111/j.1365-3156.2006.01733.x. PubMed: 17054742. 17054742

[8] Killeen GF (2013) A second chance to tackle African malaria vector mosquitoes that avoid houses and don't take drugs. Am J Trop Med Hyg 88: 809-816. doi:10.4269/ajtmh.13-0065. PubMed: 23589532. 23589532 PMC3752742

[9] Gu W, Utzinger J, Novak RJ (2008) Habitat-based larval interventions: a new perspective for malaria control. Am J Trop Med Hyg 78: 2-6. PubMed: 18187774. 18187774

[10] Kweka EJ, Zhou GF, Lee MC, Gilbreath TM, Mosha F et al. (2011) Evaluation of two methods of estimating larval habitat productivity in western Kenya highlands. Parasit Vectors 4: 110-. PubMed: 21682875. 21682875 10.1186/1756-3305-4-110 PMC3138440

[11] Mutuku FM, Alaii JA, Bayoh MN, Gimnig JE, Vulule JM et al. (2006) Distribution, description, and local knowledge of larval habitats of *Anopheles* *Gambiae* s.l. in a village in Western Kenya. Am J Trop Med Hyg 74: 44-53. PubMed: 16407345. 16407345

[12] Clark TD, Greenhouse B, Njama-Meya D, Nzarubara B, Maiteki-Sebuguzi C et al. (2008) Factors determining the heterogeneity of malaria incidence in children in Kampala, Uganda. J Infect Dis 198: 393-400. doi:10.1086/589778. PubMed: 18522503. 18522503

[13] Killeen GF, Tanner M, Mukabana WR, Kalongolela MS, Kannady K et al. (2006) Habitat targeting for controlling aquatic stages of malaria vectors in Africa. Am J Trop Med Hyg 74: 517-518. PubMed: 16606973. 16606973

[14] Smith MW, Macklin MG, Thomas CJ (2013) Hydrological and geomorphological controls of malaria transmission. Earth Science Reviews 116: 109-127. doi:10.1016/j.earscirev.2012.11.004.

[15] Githeko AK, Ototo EN, Yan GY (2012) Progress towards understanding the ecology and epidemiology of malaria in the western Kenya highlands: Opportunities and challenges for control under climate change risk. Acta Trop 121: 19-25. doi:10.1016/j.actatropica.2011.10.002. PubMed: 22015426. 22015426 PMC3298846

[16] Cohen J, Ernst C, Lindblade K, Vulule J, John C et al. (2010) Local topographic wetness indices predict household malaria risk better than land-use and land-cover in the western Kenya highlands. Malar J 9: 1-10. doi:10.1186/1475-2875-9-S1-S1. PubMed: 20043863. 21080943 PMC2993734

[17] Zhang Y, Bi P, Hiller JE (2008) Climate change and the transmission of vector-borne diseases: a review. Asia Pac J Public Health 20: 64-76. doi:10.1177/1010539507308385. PubMed: 19124300. 19124300

[18] Thomas CJ (2004) Malaria: a changed climate in Africa? Nature 427: 690-691. doi:10.1038/427690b. PubMed: 14973466. 14973466

[19] Mordecai EA, Paaijmans KP, Johnson LR, Balzer C, Ben-Horin T et al. (2013) Optimal temperature for malaria transmission is dramatically lower than previously predicted. Ecol Lett 16: 22-30. doi:10.1111/ele.12015. PubMed: 23050931. 23050931

[20] Paaijmans KP, Blanford S, Bell AS, Blanford JI, Read AF et al. (2010) Influence of climate on malaria transmission depends on daily temperature variation. Proc Natl Acad Sci U S A 107: 15135-15139. doi:10.1073/pnas.1006422107. PubMed: 20696913. 20696913 PMC2930540

[21] Paaijmans KP, Read AF, Thomas MB (2009) Understanding the link between malaria risk and climate. Proc Natl Acad Sci U S A 106: 13844-13849. doi:10.1073/pnas.0903423106. PubMed: 19666598. 19666598 PMC2720408

[22] Small J, Goetz SJ, Hay SI (2003) Climatic suitability for malaria transmission in Africa, 1911–1995. Proceedings of the National Academy of Sciences of the USA 100: 15341-15345. doi:10.1073/pnas.2236969100. 14663146 PMC307569

[23] Fillinger U, Sombroek H, Majambere S, Van Loon E, Takken W et al. (2009) Identifying the most productive breeding sites for malaria mosquitoes in The Gambia. Malar J 8: 62. doi:10.1186/1475-2875-8-62. PubMed: 19361337. 19361337 PMC2674466

[24] Majambere S, Lindsay SW, Green C, Kandeh B, Fillinger U (2007) Microbial larvicides for malaria control in The Gambia. Malar J 6: 76. doi:10.1186/1475-2875-6-76. PubMed: 17555570. 17555570 PMC1899511

[25] Do Manh C, Beebe NW, Van Nguyen Thi Van TL, Quang CTL, Van Nguyen D, et al. (2010) Vectors and malaria transmission in deforested, rural communities in north-central Vietnam. Malaria Journal 9: 259. 20846447 10.1186/1475-2875-9-259 PMC2945362

[26] Magalhães RJ, Langa A, Sousa-Figueiredo JC, Clements AC, Nery SV (2012) Finding malaria hot-spots in northern Angola: the role of individual, household and environmental factors within a meso-endemic area. Malar J 11: 1-12. doi:10.1186/1475-2875-11-S1-P1. PubMed: 22212246. 23173636 PMC3519509

[27] Bøgh C, Lindsay SW, Clarke SE, Dean A, Jawara M et al. (2007) High spatial resolution mapping of malaria transmission risk in the Gambia, West Africa, using Landsat TM satellite imagery. Am J Trop Med Hyg 76: 875-881. PubMed: 17488908. 17488908

[28] Ageep TB, Cox J, M'oawia MH, Knols BGJ, Benedict MQ et al. (2009) Spatial and temporal distribution of the malaria mosquito Anopheles arabiensis in northern Sudan: influence of environmental factors and implications for vector control. Malar J 8: 123. doi:10.1186/1475-2875-8-123. PubMed: 19500425. 19500425 PMC2698915

[29] Oesterholt MJ, Bousema JT, Mwerinde OK, Harris C, Lushino P et al. (2006) Spatial and temporal variation in malaria transmission in a low endemicity area in northern Tanzania. Malar J 5: 98. doi:10.1186/1475-2875-5-98. PubMed: 17081311. 17081311 PMC1635725

[30] Thomson MC, Mason SJ, Phindela T, Connor SJ (2005) Use of rainfall and sea surface temperature monitoring for malaria early warning in Botswana. Am J Trop Med Hyg 73: 214-221. PubMed: 16014862. 16014862

[31] Paaijmans KP, Wandago MO, Githeko AK, Takken W (2007) Unexpected high losses of Anopheles gambiae larvae due to rainfall. PLOS ONE 2: e1146. doi:10.1371/journal.pone.0001146. PubMed: 17987125. 17987125 PMC2063461

[32] Balls MJ, Bødker R, Thomas CJ, Kisinza W, Msangeni HA et al. (2004) Effect of topography on the risk of malaria infection in the Usambara Mountains, Tanzania. Trans R Soc Trop Med Hyg 98: 400-408. doi:10.1016/j.trstmh.2003.11.005. PubMed: 15138076. 15138076

[33] Bomblies A, Duchemin J-B, Eltahir EAB (2008) Hydrology of malaria: Model development and application to a Sahelian village. Water Resources Research 44: W12445.

[34] Shaman J, Stieglitz M, Stark C, Le Blancq S, Cane M (2002) Using a dynamic hydrology model to predict mosquito abundances in flood and swamp water. Emerg Infect Dis 8: 6-13. PubMed: 11749741. 11749741 10.3201/eid0801.010049 PMC2730265

[35] Charlwood JD, Vij R, Billingsley PF (2000) Dry season refugia of malaria-transmitting mosquitoes in a dry savannah zone of East Africa. Am J Trop Med Hyg 62: 726-732. PubMed: 11304064. 11304064 10.4269/ajtmh.2000.62.726

[36] Temple P, Sundborg A (1972) The Rufiji River, Tanzania hydrology and sediment transport. Geografiska Annaler 54: 345-368. doi:10.2307/520773.

[37] Charlwood JD, Kihonda J, Sama S, Billingsley PF, Hadji H et al. (1995) The rise and fall of Anopheles arabiensis (Diptera: Culicidae) in a Tanzanian village. Bulletin of Entomological Research 85: 37-44. doi:10.1017/S0007485300051993.

[38] Killeen GF, Tami A, Kihonda J, Okumu, Kotas M, et al. (2007) Cost-sharing strategies combining targeted public subsidies with private-sector delivery achieve high bednet coverage and reduced malaria transmission in Kilombero Valley, southern Tanzania. BMC Infectious Diseases 7. 10.1186/1471-2334-7-121 PMC2211306 17961211

[39] Russell TL, Govella NJ, Azizi S, Drakeley CJ, Kachur SP et al. (2011) Increased proportions of outdoor feeding among residual malaria vector populations following increased use of insecticide-treated nets in rural Tanzania. Malar J 10: 80. doi:10.1186/1475-2875-10-80. PubMed: 21477321. 21477321 PMC3084176

[40] Sinka ME, Bangs MJ, Manguin S, Rubio-Palis Y, Chareonviriyaphap T et al. (2012) A global map of dominant malaria vectors. Parasites and Vectors 5: 1-11. 22475528 10.1186/1756-3305-5-69 PMC3349467

[41] Russell TL, Lwetoijera D, Maliti D, Chipwaza B, Kihonda J et al. (2010) Impact of promoting longer-lasting insecticide treatment of bed nets upon malaria transmission in a rural Tanzanian setting with pre-existing high coverage of untreated nets. Malaria Journal 9. 10.1186/1475-2875-9-187 PMC2902500 20579399

[42] Charlwood J, Smith T, Kihonda J, Billingsley P, Takken W (1995) Density independent feeding success of malaria vectors (Diptera: Culicidae) in Tanzania. Bulletin of Entomological Research 85: 29-36. doi:10.1017/S0007485300051981.

[43] Haji H, Smith T, Charlwood JD, Meuwissen JH (1996) Absence of relationships between selected human factors and natural infectivity of Plasmodium falciparum to mosquitoes in an area of high transmission. Parasitology 113: 425-432. doi:10.1017/S0031182000081488. PubMed: 8893528. 8893528

[44] Bracken LJ, Cox NJ, Shannon J (2008) The relationship between rainfall inputs and flood generation in south–east Spain. Hydrological Processes 22: 683-696. doi:10.1002/hyp.6641.

[45] Minakawa N, Seda P, Yan G (2002) Influence of host and larval habitat distribution on the abundance of African malaria vectors in western Kenya. Am J Trop Med Hyg 67: 32-38. PubMed: 12363061. 12363061 10.4269/ajtmh.2002.67.32

[46] Minakawa N, Munga S, Atieli F, Mushinzimana E, Zhou G et al. (2005) Spatial distribution of anopheline larval habitats in Western Kenyan highlands: effects of land cover types and topography. Am J Trop Med Hyg 73: 157-165. PubMed: 16014851. 16014851

[47] Atieli HE, Zhou G, Lee M-C, Kweka EJ, Afrane Y et al. (2011) Topography as a modifier of breeding habitats and concurrent vulnerability to malaria risk in the western Kenya highlands. Parasit Vectors 4: 241. doi:10.1186/1756-3305-4-241. PubMed: 22196078. 22196078 PMC3269397

[48] Mushinzimana E, Munga S, Minakawa N, Li L, Feng C-c et al. (2006) Landscape determinants and remote sensing of anopheline mosquito larval habitats in the western Kenya highlands. Malar J 5: 1-11. doi:10.1186/1475-2875-5-1. PubMed: 16420686. 16480523 PMC1420309

[49] Nmor JC, Sunahara T, Goto K, Futami K, Sonye G et al. (2013) Topographic models for predicting malaria vector breeding habitats: potential tools for vector control managers. Parasit Vectors 6: 14 PubMed: 23324389. 23324389 10.1186/1756-3305-6-14 PMC3617103

[50] Amerasinghe PH, Amerasinghe FP, Konradsen F, Fonseka KT, Wirtz RA (1999) Malaria vectors in a traditional dry zone village in Sri Lanka. Am J Trop Med Hyg 60: 421-429. PubMed: 10466971. 10466971 10.4269/ajtmh.1999.60.421

[51] Van Der Hoek W, Konradsen F, Amerasinghe PH, Perera D, Piyaratne MK et al. (2003) Towards a risk map of malaria for Sri Lanka: the importance of house location relative to vector breeding sites. Int J Epidemiol 32: 280-285. doi:10.1093/ije/dyg055. PubMed: 12714550. 12714550

[52] Mala AO, Irungu LW, Shililu JI, Muturi EJ, Mbogo CC et al. (2011) Dry season ecology of Anopheles gambiae complex mosquitoes at larval habitats in two traditionally semi-arid villages in Baringo, Kenya. Parasit Vectors 4: 1-11. PubMed: 21352608. 21352608 10.1186/1756-3305-4-25 PMC3060147

[53] Gillies MT, De Meillon B (1968) The Anophelinae of Africa South of the Sahara. Johannesburg: South African Institute for Medical Research.

[54] Fillinger U, Kannady K, William G, Vanek MJ, Dongus S et al. (2008) A tool box for operational mosquito larval control: preliminary results and early lessons from the Urban Malaria Control Programme in Dar es Salaam, Tanzania. Malar J 7: 20. doi:10.1186/1475-2875-7-20. PubMed: 18218148. 18218148 PMC2259364

[55] Cheng P, Chaapel C ( October/November2001) Automatic DEM generation. Geoinformatics October/November: 34-39.

[56] Exelis (2012) ENVI. Version 5.0 ed. McLean, VA: Exelis Visual Information Solutions

[57] Costantini C, Li SG, Torre AD, Sagnon NF, Coluzzi M et al. (1996) Density, survival and dispersal of Anopheles gambiae complex mosquitoes in a West African Sudan savanna village. Med Vet Entomol 10: 203-219. doi:10.1111/j.1365-2915.1996.tb00733.x. PubMed: 8887330. 8887330

[58] Mwangangi JM, Shililu J, Muturi EJ, Muriu S, Jacob B et al. (2010) Anopheles larval abundance and diversity in three rice agro-village complexes Mwea irrigation scheme, central Kenya. Malar J 9: 228. doi:10.1186/1475-2875-9-228. PubMed: 20691120. 20691120 PMC2927610

[59] Scott JA, Brogdon WG, Collins FH (1993) Identification of single specimens of the Anopheles gambiae complex by the polymerase chain reaction. Am J Trop Med Hyg 49: 520–529. PubMed: 8214283. 8214283 10.4269/ajtmh.1993.49.520

[60] Koekemoer LL, Kamau L, Hunt RH, Coetzee M (2002) A cocktail polymerase chain reaction assay to identify members of the Anopheles funestus (Diptera: Culicidae) group. Am J Trop Med Hyg 66: 804-811. PubMed: 12224596. 12224596 10.4269/ajtmh.2002.66.804

[61] Bayoh MN, Akhwale W, Ombok M, Sang D, Engoki SC et al. (2011) Malaria in Kakuma refugee camp, Turkana, Kenya: facilitation of Anopheles arabiensis vector populations by installed water distribution and catchment systems. Malar J 10: 149. doi:10.1186/1475-2875-10-149. PubMed: 21639926. 21639926 PMC3130698

[62] Dongus S, Nyika D, Kannady K, Mtasiwa D, Mshinda H et al. (2009) Urban agriculture and Anopheles habitats in Dar es Salaam, Tanzania. Geospat Health 3: 189-210. PubMed: 19440962. 19440962 10.4081/gh.2009.220

[63] Ndenga BA, Simbauni JA, Mbugi JP, Githeko AK (2012) Physical, Chemical and Biological Characteristics in Habitats of High and Low Presence of Anopheline Larvae in Western Kenya Highlands. PLOS ONE 7: e47975. doi:10.1371/journal.pone.0047975. PubMed: 23110145. 23110145 PMC3479127

[64] Halekoh U, Højsgaard S, Yan J (2006) The R package geepack for generalized estimating equations. Journal of Statistical Software 15: 1-11.

[65] Canty A, Ripley B (2012) Boot: bootstrap R (S-Plus) functions. R package version 1.3-7.

[66] R Core Team (2012) R: A Language and Environment for Statistical Computing. Vienna, Austria: R Foundation for Statistical Computing.

[67] Zou GY (2008) On the estimation of additive interaction by use of the four-by-two table and beyond. Am J Epidemiol 168: 212-224. doi:10.1093/aje/kwn104. PubMed: 18511428. 18511428

[68] Smith T, Charlwood JD, Takken W, Tanner M, Spiegelhalter DJ (1995) Mapping the densities of malaria vectors within a single village. Acta Trop 59: 1-18. doi:10.1016/0001-706X(94)00082-C. PubMed: 7785522. 7785522

[69] Kiware SS, Chitnis N, Moore SJ, Devine GJ, Majambere S et al. (2012) Simplified models of vector control impact upon malaria transmission by zoophagic mosquitoes. PLOS ONE 7: e37661. doi:10.1371/journal.pone.0037661. PubMed: 22701527. 22701527 PMC3365128

[70] Kitau J, Oxborough RM, Tungu PK, Matowo J, Malima RC et al. (2012) Species shifts in the Anopheles gambiae complex: do LLINs successfully control Anopheles arabiensis? PLOS ONE 7: e31481. doi:10.1371/journal.pone.0031481. PubMed: 22438864. 22438864 PMC3306310

[71] Okumu FO, Mbeyela E, Lingamba G, Moore J, Ntamatungiro AJ et al. (2013) Comparative field evaluation of combinations of long-lasting insecticide treated nets and indoor residual spraying, relative to either method alone, for malaria prevention in an area where the main vector is Anopheles arabiensis. Parasites and Vectors 6: 46. 23433393 10.1186/1756-3305-6-46 PMC3606331

[72] Govella NJ, Chaki PP, Killeen GF (2013) Entomological surveillance of behavioural resilience and resistance in residual malaria vector populations. Malar J 12: 124. doi:10.1186/1475-2875-12-124. PubMed: 23577656. 23577656 PMC3637503

[73] Kiszewski A, Mellinger A, Spielman A, Malaney P, Sachs SE et al. (2004) A global index representing the stability of malaria transmission. Am J Trop Med Hyg 70: 486-498. PubMed: 15155980. 15155980

[74] Gimnig JE, Ombok M, Kamau L, Hawley WA (2001) Characteristics of larval anopheline (Diptera: Culicidae) habitats in Western Kenya. J Med Entomol 38: 282-288. doi:10.1603/0022-2585-38.2.282. PubMed: 11296836. 11296836

[75] Tuno N, Githeko A, Yan G, Takagi M (2007) Interspecific variation in diving activity among *Anopheles* *gambiae* Giles, An. arabiensis Patton, and An. funestus Giles (Diptera: Culicidae) larvae. Journal of Vector Ecology 32: 112-117. 17633431 10.3376/1081-1710(2007)32[112:ividaa]2.0.co;2

[76] Jacob BG, Arheart KL, Griffith DA, Mbogo CM, Githeko AK et al. (2005) Evaluation of environmental data for identification of Anopheles (Diptera: Culicidae) aquatic larval habitats in Kisumu and Malindi, Kenya. J Med Entomol 42: 751–755. Available online at: doi:10.1603/0022-2585(2005)042[0751:EOEDFI]2.0.CO;2. PubMed: 16365996 16365996 10.1093/jmedent/42.5.751 PMC2673498

[77] Dukeen MY, Omer S (1986) Ecology of the malaria vector Anopheles arabiensis Patton(Diptera: Culicidae) by the Nile in northern Sudan. Bulletin of Entomological Research 76: 451-467. doi:10.1017/S0007485300014942.

[78] Shousha AT (1948) Species-eradication: The Eradication of Anopheles gambiae from Upper Egypt, 1942-1945. Bull World Health Organ 1: 309–352. PubMed: 20603927. 20603927 PMC2553915

[79] Himeidan YE, Elzaki MM, Kweka EJ, Ibrahim M, Elhassan IM (2011) Pattern of malaria transmission along the Rahad River basin, Eastern Sudan. Parasit Vectors 4: 1-9. PubMed: 21679459. 21679459 10.1186/1756-3305-4-109 PMC3128851

[80] Craig M, Snow R, Le Sueur D (1999) A climate-based distribution model of malaria transmission in sub-Saharan. Africa - Parasitology Today 15: 105-111. doi:10.1016/S0169-4758(99)01396-4. 10322323

[81] Hoshen MB, Morse AP (2004) A weather-driven model of malaria transmission. Malar J 3: 32. doi:10.1186/1475-2875-3-32. PubMed: 15350206. 15350206 PMC520827

[82] Thomson MC, Doblas-Reyes FJ, Mason SJ, Hagedorn R, Connor SJ et al. (2006) Malaria early warnings based on seasonal climate forecasts from multi-model ensembles. Nature 439: 576-579. doi:10.1038/nature04503. PubMed: 16452977. 16452977

