# Supplementary material for: Habitat Hydrology and Geomorphology Control the Distribution of Malaria Vector Larvae in Rural Africa
Source: PLoS One. 2013 Dec 3;8(12):e81931. doi: 10.1371/journal.pone.0081931 (PMC3849348; doi:10.1371/journal.pone.0081931)
Supplement: Methods S1 — Description of the statistical analysis of entomological data: Generalized Estimating Equations and Method of Variance Estimate Recovery. (DOCX) [file pone.0081931.s001.docx]

# Supplementary Methods S1

# “Habitat hydrology and geomorphology control the distribution of malaria vector larvae in rural Africa”

Andrew J Hardy^1,2^, Javier G P Gamarra^3^, Dónall E Cross^2,3^, Mark G Macklin^1^, Mark W Smith^4^, Japhet Kihonda^2^, Gerry F Killeen^2,5^, George N Ling’ala^2^, Chris J Thomas^3*^.

1 Institute of Geography & Earth Sciences, Aberystwyth University, Aberystwyth, UK.

2 Biomedical and Environmental Sciences Thematic Group, Ifakara Health Institute, Ifakara, Tanzania.

3 Institute of Biological, Environmental and Rural Sciences, Aberystwyth University, Aberystwyth, UK.

4 School of Geography, University of Leeds, Leeds, UK.

5 Vector Biology Department, Liverpool School of Tropical Medicine, Liverpool, UK.

*Corresponding author. Tel: +44 1970 621 987, email: cjt@aber.ac.uk

## Statistical analysis of entomological data

#### Computation of the number of Anopheles arabiensis larvae

Given the highly heterogeneous nature of the data collected, a semiparametric approach that provides robust estimates in the presence of unknown of misspecified covariance structures. In this case we chose Generalized Estimating Equations (GEEs) as opposed to General Linear Mixed Models.

All GEEs were clustered by site, and corrected with sandwich estimators for the standard errors. Quasi-likelihood estimators are produced via iterative algorithms. Two independent GEE models were computed:

1. A GEE modelling the total number of anophelines per dip taken in the field. The family distribution of the larvae was assumed to be Poisson with a log link.
2. A GEE modelling the proportion of PCR samples (a subsample from the total anophelines in the field) that correspond to *An. arabiensis*. The family distribution of the proportion was assumed to be binomial with a logit link.

The product of the two GEE estimators (number of anopheline larvae and fraction of them that corresponds to *An. arabiensis*) is the estimated number of *An. arabiensis* taken in the field. To compute it, a mixture Poisson-binomial distribution was produced with the means and confidence intervals obtained in the previous GEEs. For example, assume two random variables: anopheline larvae *L* and the fraction of those that are *An. arabiensis* (*F*) following Poisson (λ) and binomial (*p*) distributions respectively. The expectation for the number of *An. arabiensis* in the sample is:

$$E_{arab}=E\left( E\left( F|L \right) \right)=E\left( pL \right)=p\lambda$$

Via optimization procedures we calculated the number of events necessary to simulate both the Poisson and binomial distributions with the same confidence intervals as those resulting from the GEE. After stochastic generation, the distributions were mixed.

Next, we bootstrapped the resulting mixture 100 times to calculate the first moment and confidence intervals of the mixture distribution, providing the estimate number of *An. arabiensis* per dip and the corresponding confidence interval.

#### Contrasts via Method of Variance Estimate Recovery (MOVER)

Once obtained the robust estimators and their confidence intervals, overlaps of the differences between estimators were obtained by pairwise contrasts. The MOVER method [[1](#_ENREF_1),[2](#_ENREF_2),[3](#_ENREF_3)] provides a methodology to calculate upper and lower overlaps between confidence intervals and their significance. Assuming 95% two-sided confidence intervals for estimates, the lower and upper limits *D_l_* and *D_u_* for the difference between two log-transformed parameter estimates *β_1_* and *β_2_* are:

$$D_{i}=\left( \beta_{1}+\beta_{2} \right)-z_{0.05/2}\sqrt{\mathrm{var}\left( \beta_{1} \right)+\mathrm{var}\left( \beta_{2} \right)}$$

$$D_{u}=\left( \beta_{1}+\beta_{2} \right)-z_{0.05/2}\sqrt{\mathrm{var}\left( \beta_{1} \right)+\mathrm{var}\left( \beta_{2} \right)}$$

where *z_0.05/2_* is the upper 95% quantile of a standard normal distribution.

## References

1. Zou GY (2008) On the estimation of additive interaction by use of the four-by-two table and beyond. The American Journal of Epidemiology 168: 212-224.

2. Zou G, Donner A (2008) Construction of confidence limits about effect measures: a general approach. Statistics in Medicine 27: 1693-1702.

3. Kowalik D, Choi Y-H, Zou G (2011) Confidence interval estimation for a difference between two dependent intraclass correlation coefficients with variable class sizes. Journal of Statistical Theory and Practice 5: 613-625.
